# Amidoxime Polymers for Uranium Adsorption: Influence of Comonomers and Temperature

**DOI:** 10.3390/ma10111268

**Published:** 2017-11-04

**Authors:** Austin P. Ladshaw, Alexander I. Wiechert, Sadananda Das, Sotira Yiacoumi, Costas Tsouris

**Affiliations:** 1 School of Civil and Environmental Engineering, Georgia Institute of Technology, Atlanta, GA 30332, USA; aladshaw3@gatech.edu (A.P.L.); awiechert3@gatech.edu (A.I.W.); sotira.yiacoumi@ce.gatech.edu (S.Y.); 2 Oak Ridge National Laboratory, Oak Ridge, TN 37831, USA; amisdas@gmail.com

**Keywords:** uranium adsorption, amidoxime, comonomer, seawater, temperature effect, modeling

## Abstract

Recovering uranium from seawater has been the subject of many studies for decades, and has recently seen significant progress in materials development since the U.S. Department of Energy (DOE) has become involved. With DOE direction, the uranium uptake for amidoxime-based polymer adsorbents has more than tripled in capacity. In an effort to better understand how these new adsorbent materials behave under different environmental stimuli, several experimental and modeling based studies have been employed to investigate impacts of competing ions, salinity, pH, and other factors on uranium uptake. For this study, the effect of temperature and type of comonomer on uranium adsorption by three different amidoxime adsorbents (AF1, 38H, AI8) was examined. Experimental measurements of uranium uptake were taken in 1−L batch reactors from 10 to 40 °C. A chemisorption model was developed and applied in order to estimate unknown system parameters through optimization. Experimental results demonstrated that the overall uranium chemisorption process for all three materials is endothermic, which was also mirrored in the model results. Model simulations show very good agreement with the data and were able to predict the temperature effect on uranium adsorption as experimental conditions changed. This model may be used for predicting uranium uptake by other amidoxime materials.

**Notice:** This manuscript has been authored by UT-Battelle, LLC under Contract No. DE-AC05-00OR22725 with the U.S. Department of Energy (DOE). The United States Government retains and the publisher, by accepting the article for publication, acknowledges that the United States Government retains a non-exclusive, paid-up, irrevocable, world-wide license to publish or reproduce the published form of this manuscript, or allow others to do so, for United States Government purposes. The Department of Energy will provide public access to these results of federally sponsored research in accordance with the DOE Public Access Plan (http://energy.gov/ downloads/doe-public-access-plan).

## 1. Introduction

### 1.1. Background

At the current rate of consumption, conventional terrestrial reserves of uranium will be depleted within the next 100 years [1]. If nuclear energy is to remain a sustainable energy source or be used to reduce greenhouse gas emissions, then a long term and economically competitive supply of uranium must be accessed. Seawater, which contains a reserve of approximately 4.5 billion metric tons of dissolved uranium, is one potential uranium resource [2]. This vast reserve would ensure a centuries-long uranium supply, provide a cap on the price of uranium, and allow for increased production of nuclear energy to reduce greenhouse gas emissions. The recovery of uranium from seawater is, however, a challenging task due to the low concentration of uranium (~3.3 ppb) and the numerous competing ions in seawater such as calcium, magnesium, and particularly vanadium. Furthermore, the species of uranium in seawater, which consist primarily of very stable uranyl-carbonate complexes, make recovery even more challenging [2]. Thus, any adsorbent used for uranium recovery from seawater not only must be highly selective, efficient, and reusable but must also be able to compete with the carbonate to form complexes with uranyl.

Over the past 60 years, numerous studies have been performed in search of an appropriate adsorbent for the recovery of uranium from seawater. Various materials such as metal adsorbents, organic resins, and biosorbents have been considered, though amidoxime based adsorbents are considered to be the most promising [1,3,4,5]. Development of amidoxime adsorbents in Japan during the 1980s, led to several marine tests in the 1990s and early 2000s that culminated in the successful recovery of over 1 kg of uranium using passive amidoxime adsorbents that take advantage of the continuous motion of the ocean water [1,6,7]. Since then, the U.S. Department of Energy (DOE) has sought to continue research into uranium recovery by supporting multidisciplinary research into materials development, advanced characterization, and the development of high performance computational models [8]. DOE support of amidoxime research has, in turn, led to the development of materials with more than three times the adsorption capacity of the Japanese materials, increasing from 1.8 mg U/g adsorbent to 6.56 mg U/g adsorbent after 56 days of exposure [9].

### 1.2. Review of Materials Tested for Uranium Adsorption

#### 1.2.1. Non–Amidoxime Materials

Since the 1950s, the recovery of uranium from seawater has been the subject of numerous studies examining a wide variety of adsorbents, ion exchangers, and biosorbents. Some of the adsorbents included zinc carbonate [10], lead sulfide [10,11,12] (galena), titanic acid [12], activated carbon [13], iron (III) oxide [14], and hydrous titanium oxide (TiO_2_) [10,11,13,14]. Galena and TiO_2_ with uranium adsorption capacities up to 1.1 and 0.6 mg U/g adsorbent, respectively, were considered to be the most promising of these adsorbents. Despite galena having a higher potential adsorption capacity, later tests showed that keeping the surface of the adsorbent fresh was difficult without reducing the adsorption capacity [3]. Thus, among the materials introduced in the literature for uranium recovery from seawater, TiO_2_ received the most significant amount of attention until the application of amidoxime [1,3,4]. Organic ion-exchange resins such as Zeo-Karb 226, 8-hydroxyquinoline, and resorcinol arsenic acid were also considered, though these materials either deteriorated rapidly [4] or had low uranium selectivity [5]. Dithiocarbamate chelating resin was another more promising ion-exchanger with a one-day adsorption of 50 µg U/g adsorbent and an estimated capacity of 5.1 mg U/g adsorbent [15]. It does not appear, however, that the material was developed further for uranium recovery from seawater.

In addition to the various materials discussed previously, a number of recovery techniques have also been considered. Actively pumped fixed bed systems were initially considered to be the most realistic method for uranium recovery from seawater [3,16,17]. The construction, maintenance and operating costs associated with such a facility, however, greatly reduce its economic competitiveness. Potential material and eluent losses also add to the cost of actively pumped systems. Activated carbon or carbon composites can be used to eliminate elution costs by burning the adsorbent after the first loading, though this would only be economical if the activated carbon could be obtained cheaply [13]. Along with its relatively low recovery rates, solvent extraction [18,19] is further harmed by solvent losses through entrainment and dissolution, making the process cost prohibitive [4]. Despite having higher recovery rates, flotation techniques [20,21] are undesirable due to surfactant losses [9] and the time needed to obtain high recovery rates [3]. Compared to the previously mentioned techniques, the application of magnetic adsorption [14] to uranium recovery from seawater has received relatively little attention, though recovery from other aqueous solutions has been reported [22,23].

Biosorbents including an array of microorganisms and biomolecules have been and continue to be examined including various species of freshwater algae [24], cyanobacteria [25], fungi [26], engineered proteins [27], plant wastes [28], and immobilized tannins [29,30]. Biomaterials have a number of advantages over the other materials discussed previously; their principal advantages being that they are both renewable and do not negatively impact the environment, though biomaterials tend to be more sensitive to environmental factors such as pH and temperature. High uranium selectivity is another potential advantage of these materials. Immobilized chestnut skins are one example, allowing 80% uranium recovery in seawater, while tannin crosslinked on polyvinyl alcohol and cyanobacteria also achieved reasonable recovery rates of 2.5 and 3.0 mg U/g adsorbent, respectively. Furthermore, our ability to develop mutant or engineered proteins, such as the protein prepared by Zhou [23] and coworkers using a protein from the methanogen Methanobacterium thermoautotrophicum, allows for further selectivity enhancements. It should also be noted that adsorption capacity and uranium selectivity are generally independent of microbial life functions [5]. This is potentially advantageous as it removes any need for an input of nutrients into the recovery system.

#### 1.2.2. Japanese Amidoxime Adsorbents

The Japanese began development of amidoxime adsorbents for the recovery of uranium from seawater in the late 1970s and early 1980s. Among the earliest to study amidoxime for uranium recovery was Sugasaka and coworkers [31] who synthesized amidoxime chelating resins with various copolymers and crosslinking agents with a maximum adsorption of 3.2 mg U/g adsorbent after 180 days in seawater at room temperature. Later, Kobuke and coworkers [32] developed an amidoxime-silica composite fiber, where powdered adsorbent was entrapped into a support material of polyethylene or an ethylene-vinyl acid copolymer, with an uptake of 0.2 mg U/g adsorbent after one day in a seawater batch test. During this time period, amidoxime adsorbents such as amidoxime based polymer beads, chemically prepared amidoxime fiber, and fibers prepared by radiation induced graft polymerization (RIGP) were developed for practical applications in seawater [6]. Amidoxime polymer beads were prepared by synthesizing and then amidoximating polymer beads containing amidoxime precursor cyano groups. Furthermore, polymer beads required packaging to ensure effective contact with seawater. Chemically prepared fibers were amidoximated from commercially available acrylonitrile fibers; however, the even distribution of amidoxime groups in the fiber led to a loss of mechanical strength after amidoximation. To improve the mechanical strength, a high strength trunk fiber was used with RIGP to graft amidoxime groups to the surface of the trunk fiber.

Marine testing of amidoxime began in the early 1990s with the work of Takeda and coworkers. Takeda used a fixed bed of hollow fibers containing amidoxime groups prepared via RIGP and obtained 0.97 mg U/g adsorbent after 30 days of contact with coastal seawater at an average superficial velocity of 4 cm/s [33]. Shortly thereafter, Egawa and coworkers examined the uptake of a high porosity amidoxime resin prepared from acrylonitrile-divinylbenzene copolymer beads, through various mooring and towing trials [34]. For both sets of trials, beds of amidoxime resin were suspended in the ocean from a buoy. The highest uranium uptake rate of 1.32 mg U/g adsorbent was obtained after mooring the adsorbent for 830 hours (34.5 days). A similar experiment was performed at the end of the decade by Seko and coworkers [7] using polyethylene/polypropylene nonwoven fabric grafted with amidoxime packed into cages and suspended in the ocean. This experiment yielded over one kilogram of uranium, roughly 2.85 mg U/g adsorbent, after 240 days of contact with seawater. Of particular interest to this work was the study performed by Shimizu and Tamada [35]. For their study, amidoxime fibers braided around a polypropylene trunk material were anchored to the bottom of the sea off the coast of Okinawa. The uranium uptake of these fibers was 1.5 mg U/g adsorbent after a contact period of 30 days.

#### 1.2.3. ORNL Amidoxime Adsorbents

In recent years, under the direction of DOE, Oak Ridge National Laboratory (ORNL) has continued the development of amidoxime adsorbents that were pioneered by the Japanese in the early 2000s. The ORNL amidoxime adsorbents were first tested by Kim and coworkers [36] in batch and flow–through column experiments performed with seawater taken 75 miles east of Savannah, Georgia, and near shore in Charleston, South Carolina. For the flow-through experiments, amidoxime-methacrylic acid copolymers were grafted to a polyethylene (PE) support matrix by RIGP. That adsorbent material formulation was denoted as the 38H formulation and was packed into a number of columns for seawater capacity testing. The 38H packed columns were compared against columns containing nonwoven PE sheets provided by the Japanese Atomic Energy Agency (JAEA). The uranium uptake rate of the ORNL adsorbents from both seawater sources was about 3.3 mg U/g after a period of 56 days, which was roughly three times higher than that of the JAEA reference materials. A follow-up study using the same materials and performed at the Marine Sciences Laboratory of the Pacific Northwest National Laboratory (PNNL) with seawater from the Sequim Bay yielded similar results [37].

To study the effects of polymer composition and conditioning, studies were performed by Das and coworkers using new material formulations: ORNL’s AI (amidoxime-vinylphosphonic acid copolymer) [38] and AF (amidoxime-itaconic acid copolymer) [39] series adsorbents. The AI and AF adsorbents, made from hollow gear shaped PE fibers with various amidoxime to acid comonomer mole ratios, were tested in flow-through columns at PNNL. Both series were conditioned in 0.44 M potassium hydroxide (KOH) at 80 °C for time periods of either 1 hour or 3 h. Of these two series, AF adsorbents with an amidoxime-to-itaconic acid molar ratio of about 10.14 and 1 h KOH conditioning achieved the highest uptake rate of 3.9 mg U/g adsorbent in 56 days. The highest recovery rate for the AI series, 3.35 mg U/g adsorbent, was obtained with an amidoxime-to-vinylphosphonic acid molar ratio of 3.52 after 3 h of KOH conditioning.

A modification made by ORNL to the adsorbent synthesis was the use of atom transfer radical polymerization (ATRP) to replace RIGP for grafting adsorbents to the support polymers. ATRP has a number of advantages over RIGP including greater control over polymer composition, conformation, morphology, and significantly reduced homopolymer formation. This, in turn, allows for easier manipulation of polymer molecular weight, weight distribution, and improved synthesis of block and graft copolymers [40]. The application of ATRP to amidoxime polymer synthesis was investigated by Brown and coworkers using an amidoxime-acrylic acid copolymer grafted to Poly (vinyl chloride)-co-chlorinated Poly (vinyl chloride) fibers [40]. Flow-through column experiments showed a uranium recovery rate of 6.56 mg U/g adsorbent after 56 days in contact with seawater, which is the highest adsorption capacity observed to date [9]. The use of alternative alkaline conditioners, such as sodium hydroxide (NaOH), has also been considered to replace KOH conditioning. A study by Das and coworkers [41] showed that amidoxime polymers conditioned with NaOH and KOH had similar adsorption capacities, though the cost of NaOH is significantly less.

### 1.3. Focus of This Study

Since the DOE’s involvement in the investigation of uranium recovery from seawater, the primary research focus has been on the development of new adsorbent materials that could be highly selective for uranium and produce higher uranium capture capacities [36,38,39,42]. To supplement this effort, universities and laboratories around the country [43,44,45,46] have spent a great deal of effort trying to understand how the chemisorption of uranium occurs and the environmental factors that affect uranium capture efficiency and cost. To this end, both experimental and modeling studies have been performed to quantify effects such as competing metal ions for amidoxime sites [47,48,49], salinity and pH impacts on uranium capacity [2,50], and the influence of seawater velocity on mass transfer resistances for braided fiber adsorbents [51]. In this study, the focus is on how the seawater temperature can impact uranium chemisorption by the ORNL adsorbent materials and how those impacts can be modeled to accurately predict the uranium adsorption capacity.

Adsorption experiments with three different adsorbents (38H, AI8, and AF1) are examined to quantify the impact of temperature on uranium uptake capacities. While these materials have essentially the same types of active surface ligands for capturing uranium, prior studies have noted that they have different uranium capacities from both laboratory and seawater experiments [9,37,38,39]. The major difference between these materials is the acid comonomers used in the grafting of the ligands. Until now, there has been little effort made towards understanding the mechanism that contributes to these differences in adsorption capacity by changing the comonomers. Therefore, as an additional part of this study, a chemisorption model is developed to account for the impact of the comonomer grafted to the materials. This model is then utilized to predict material performance for all three adsorbents across a range of temperatures relevant to seawater. As this model includes the impact of comonomers on chemisorption of uranium, it could then potentially also be used as a tool to determine what types of comonomers may result in optimal uranium uptake.

## 2. Materials and Methods

### 2.1. Adsorbent Materials

The adsorbents examined in this study were the 38H, AI8, and AF1 materials developed by ORNL [38,39,52,53]. These materials are formed from high-surface-area hollow gear, polyethylene (PE) fibers grafted with polyacrylonitrile (AN) and various comonomers using RIGP. Note that the PE fibers used in this study have an approximate surface area of 1.35 m^2^/g [54]. Each formulation has a different comonomer: 38H uses methacrylic acid (MAA), AI8 uses vinylphosphonic acid (VPA), and AF1 uses itaconic acid (ITA). Subsequently, each of the fibers was amidoximated with hydroxylamine in a methanol/water mixture, whereby the nitrile group was converted to amidoxime. Prior to use in adsorption experiments, the fibers were conditioned with 0.44 M KOH at 80 °C for 3 h. Additional details regarding these materials are described by Kuo et al. [49], Gill et al. [43], and Das et al. [38,39].

Elemental analyses have been performed on the 38H [52] and AF1 [39], both before and after the grafting of the AN and comonomers, as well as after amidoximation and conditioning. Results from the elemental analysis for 38H are shown in Table 1 below. Note that the elemental analysis results for AF1 have been previously published by Das et al. [39]. For the AI8 material, no elemental analysis was performed; however, the degree of grafting (DOG) was estimated by measuring the dry weight of the fiber before and after the grafting of AN and VPA. AI8 had a DOG of roughly 269% in a graft solution with an AN/VPA molar ratio of 3.52 [38]. It is worth noting that the AN-to-comonomer molar ratio found on the 38H and AF1 fibers was slightly different from that ratio in the graft solution [39,52]. Therefore, the AN/VPA molar ratio in the graft solution is not necessarily the same as the AN/VPA ratio that is actually grafted onto the fiber.

Based on Table 1 and the elemental analysis of AF1 by Das et al. [39], it is found that prior to grafting of the copolymers, the PE fiber contains essentially no nitrogen (N). In addition, since neither of the comonomers (MAA and ITA) contains N atoms, it can be assumed that all N atoms gained by the fibers post-grafting are in the form of AN. After amidoximation with hydroxylamine, some additional N atoms are gained. Then, from that weight percent. the total N concentration on the 38H and AF1 fibers can be determined (Table 2). For instance, a post-amidoximation N wt % of 13.8 would lead to a molar N concentration of about 9.9 mol/kg after multiplying N wt % by 10^3^ to convert to g/kg and then dividing by the atomic weight of N (14 g/mol).

The approximation of the molar concentration of the comonomers is then based on the weight of oxygen (O) gained and the molar ratio of N/O after grafting. Using the pre-amidoximation weight is necessary because, after the amidoximation step, there are additional O atoms gained that are not associated with the comonomer. Before amidoximation, it can be assumed that all O atoms gained are from the comonomer and then, depending on the number of O atoms associated with the particular comonomer of interest, the molar concentration of the comonomers can be determined for each material. A summary of this analysis is available in Table 2.

### 2.2. Functional Surface Groups

For each adsorbent material, there are three different surface groups that are of interest: the acidic comonomer and two different amidoxime ligands. While each material has a different comonomer, they all have the same amidoxime ligands, which are the active chemisorption sites. The active amidoxime surface sites have been previously identified as an open-chain amidoxime resembling acetamidoxime (AO) and a cyclic amidoxime that resembles glutarimide-dioxime (IDO). Although other amidoxime ligands may also form in the polymer chains, these two are believed to be the most prevalent ligands formed during the amidoximation process [55].

From the N concentration on each material (Table 2), a formula can be derived to determine how much of each ligand (*L*_max,AO_ and *L*_max,IDO_ in mol/kg) can be present on the fiber (Equation (1)). This equation is based on the assumption that all N atoms on the fiber (*T*_N_ in mol-N/kg) are either forming the AO or IDO ligands and the fact that AO contains 2 mol-N/mol-AO and IDO has 3 mol-N/mol-IDO. In a previous analysis of the AF1 materials, it was found that these fibers contained approximately 1.45 mol-AO/kg-fiber and 3.3 mol-IDO/kg-fiber [56]. The ratio of AO/IDO ligands for 38H and AI8, however, is unknown:

(1) $$T_{N}=2L_{\max,\mathrm{AO}}+3L_{\max,\mathrm{IDO}}.$$

### 2.3. Experimental Setup

All adsorption experiments were performed using 1-L plastic bottles as batch reactors, placed in a temperature-controlled shaker (MaxQ 4000, Thermo Fisher Scientific, Marietta, OH, USA). The shaker was set at 100 rpm. Each batch solution was prepared using 193 ppm sodium-bicarbonate (NaHCO_3_) to emulate the carbonate concentration of seawater, 0.43 M sodium-chloride (NaCl) to simulate the salinity of seawater, and roughly 7.5 ppm of uranium as uranyl-nitrate [UO_2_(NO_3_)_2_] to provide a sufficient uranium concentration for uptake and capacity measurements. This is the same formulation of the brine solution used in previous uranium studies [36,37,38,39] and includes an appropriate level of uranium for chemical analyses using inductively coupled plasma optical emission spectroscopy (ICP OES, Perkin Elmer Optima 2100DV, Wellesley, MA, USA). The pH of each solution was relatively constant at approximately 7.5±0.1 throughout the duration of each experiment. The temperature for each experiment was held constant throughout the duration of the experiment, but varied from 10 °C to 40 °C for different experimental runs.

Solution samples were obtained periodically from each batch to analyze the concentration of uranium. The analysis was performed by ICP OES (Perkin Elmer, Wellesley, MA, USA). At each time step, the drop in aqueous uranium concentration measured was used to estimate the amount of uranium taken up by the fibers. Once the aqueous uranium concentration was no longer changing, or only slightly changing, the experiment was stopped and a final estimate for uranium adsorption capacity was extrapolated and averaged from the data collected. That final estimate of the uranium capacity was then considered as the equilibrium chemisorption capacity for the experimental run.

The 38H and AI8 materials were tested using roughly 15 mg of adsorbent for each experimental run. For the AF1 material, two fibers were examined: AF160 and AF1FR3. These AF1 fibers were each prepared in an identical fashion, but produced and tested at different times. As such, they are treated as the same material for the purpose of this study. The AF160 was tested using 15 mg of fiber and the AF1FR3 was tested at 15, 10, and 5 mg of fiber.

### 2.4. Modeling Methods

To adequately model the complexity of this experimental system, the model must be constructed such that the effects of ionic strength, aqueous speciation, salinity, pH, and temperature can be implicitly included in the calculation of uranium chemisorption. To this end, a modeling approach similar to that of the MINEQL software [57,58] has been adopted. The modeling structure that has been developed for this work is an object-oriented software framework whereby different modules or objects can be added to represent different bits of chemistry or physics or removed to change the specificity of the problem being solved. Then, the culmination of all those modules creates a single common residual function that can be used to iteratively and simultaneously solve the system of equations for all unknowns [2,56]. Additional information on this modeling architecture is provided in Supplemental Information.

A significant portion of the model is made up of aqueous equilibrium reactions in order to depict the speciation that occurs in the solution phase of the experiments. As with all equilibrium reactions, the equilibrium constant (*K_i_*) is inherently a function of temperature through the van't Hoff relationship (Equation (2)). In van't Hoff's equation, *K_i_* is calculated from the gas law constant (*R*), the system temperature (*T*), and the Gibb's free energy of the reaction (Δ*G_i_*). In turn, the free energy parameter may also vary with temperature based on the enthalpy (Δ*H_i_*) and entropy (Δ*S_i_*) of the reaction (Equation (3)). Those reaction energies can be estimated based on the stoichiometric coefficients (*υ_i_*) of the reaction and the formation energy terms (*G_f,i_^o^*, *H_f,i_^o^*, and *S_i_^o^*) for each aqueous species (Equation (4)) [59,60,61]. A table of the formation energy terms and aqueous reactions employed by this model is available in Supplemental Information. Note that, when enthalpy and entropy terms for any reaction or species were unknown or unavailable, the Gibb's free energy term was used instead:

(2) $$\ln K_{i}=-\frac{\Delta G_{i}}{RT},$$

(3) $$\Delta G_{i}=\Delta H_{i}-T\Delta S_{i},$$

(4) $$\begin{array}{l} \Delta H_{i}=\sum_{\mathrm{products}}\upsilon_{i}H_{f,i}^{o}-\sum_{\mathrm{reactants}}\upsilon_{i}H_{f,i}^{o}\\ \Delta S_{i}=\sum_{\mathrm{products}}\upsilon_{i}S_{i}^{o}-\sum_{\mathrm{reactants}}\upsilon_{i}S_{i}^{o}\\ \Delta G_{i}=\sum_{\mathrm{products}}\upsilon_{i}G_{f,i}^{o}-\sum_{\mathrm{reactants}}\upsilon_{i}G_{f,i}^{o} \end{array}$$

Along with the aqueous reactions are the chemisorption reactions that occur with each surface ligand. These site-specific interactions can be represented mathematically as shown in Equation (5). In this generalized expression, the specific adsorbate is identified by the *i* subscript, while other aqueous ions involved in the reaction are denoted by *j* and *k* subscripts. Activities of the participating aqueous ion are denoted by curly bracket notation (i.e., {*i*} is the activity of the *i*^th^ aqueous species). The subscript *l* denotes a different ligand for each reaction, which can involve different numbers of sites (*m_i,l_*) for the adsorbate/ligand pair. The molar concentration of available sites for the *l*^th^ ligand is represented by [*L*ϕ]*_l_*, and {*q_i,l_*} represents the chemisorption activity of the *i*^th^ adsorbate to that site [60,62]. Note that the subscripts of *i,l* refer to the *i*^th^ adsorbate that is bonded with the *l*^th^ ligand:

(5) $$\upsilon_{i}\left\{i\right\}+\sum_{j}\upsilon_{j}\left\{j\right\}+m_{i,\;l}{\left[L\phi \right]}_{l}\Leftrightarrow \left\{q_{i,\;l}\right\}+\sum_{k}\upsilon_{k}\left\{k\right\}.$$

The concentration of surface ligands that are available to react with an adsorbate is a function of the maximum surface density of that ligand (*L*_max,*l*_) and the sum of adsorbed species occupying those specific sites (Equation (6)). As with aqueous reactions, the equilibrium constants associated with chemisorption reactions (*K_i_*_,*l*_) are also related to temperature through the van't Hoff expression (Equation (2)). The total equilibrium expression for chemisorption, however, is further tied to temperature via the surface activity parameter (*γ* ^s^*_i_*_,*l*_) and the Boltzmann factor (*η_i,l_*), which is used to adjust the equilibrium coefficient based on the accumulation of surface charge (Equations (7) and (8)) [61,62]. Additional information on surface charging and surface activity, as well as definitions for the parameters of the Boltzmann factor are available in Supplemental Information:

(6) $${\left[L\phi \right]}_{l}=L_{\max,\;l}-\sum_{\forall i\;\in \;l}m_{i,\;l}q_{i,\;l},$$

(7) $$K_{i,\;l}\cdot \eta_{i,\;l}=\frac{\left(\gamma_{i,\;l}^{s}q_{i,\;l}\right)\cdot \prod {\left\{k\right\}}^{\upsilon_{k}}}{{\left[L\phi \right]}_{l}^{m_{i,\;l}}\cdot {\left\{i\right\}}^{\upsilon_{i}}\cdot \prod {\left\{j\right\}}^{\upsilon_{j}}},$$

(8) $$\eta_{i,\;l}=\exp\left(-\frac{N_{i,\;l}e\psi }{k_{B}T}\right).$$

### 2.5. Relevant Ligand and Comonomer Reactions

The AO and IDO ligands attract uranium from solution by reacting with uranyl (UO_2_^2+^) ions in solution. A set of 12 chemisorption reactions (5 for AO and 7 for IDO) has been proposed as the primary chemisorption reactions between uranyl and amidoxime on the surface of the ORNL adsorbent materials based on their relative binding affinities (Table 3) [47,56,63,64,65]. From previous simulations using these reactions, it has been seen that three species [UO_2_(AO)_2_, UO_2_(H_2_IDO)(HIDO)^−^, and UO_2_(H_2_IDO)_2_] contribute to over 99% of all adsorbed uranium on AF160 fibers near neutral pH (7 to 8) [56]. Note that the enthalpy and entropy of reaction for UO_2_(AO)_2_ was determined iteratively in this study (see Results and Discussion).

While the chemisorption reactions in Table 3 are the most important reactions and are directly related to uranium chemisorption, the protonation and/or deprotonation of the ligand and comonomers can have an effect on chemisorption as well. The p*K_a_*s for the ligands measure how strongly bound protons are to the ligands and because the uranyl ions must displace the protons in order to bind (Table 3), those p*K_a_*s can play a vital role in the overall chemisorption strength for each reaction [52,53,63]. Additionally, the protonation and/or deprotonation of both the ligands and the comonomers will inevitably affect the accumulation of surface charge for the adsorbent. Therefore, the comonomers themselves may have an indirect impact on uranium chemisorption through surface charging [60,62,67]. Thus, these reactions should also be considered in the chemisorption model. A summary of the p*K_a_*s for the ligands and comonomers is provided in Table 4.

## 3. Results and Discussion

### 3.1. Optimization of AO Reaction with AF160 Material

The enthalpies and entropies of the reactions between IDO ligands and uranyl ions have been determined experimentally and are available from literature reports (Table 3) [65]. These values can be used directly in the chemisorption model to simulate the temperature effect on uranyl uptake by IDO ligands. Enthalpies and entropies for the AO reactions, however, are unknown. Therefore, modeling of the temperature effect of uranium chemisorption by AO ligands will require some preliminary data analysis.

Table 3 shows that there are five different AO chemisorption reactions that the model will consider when simulating uptake. Of those five reactions, it has been previously noted that only the reaction forming the UO_2_(AO)_2_ species shows any appreciable level of chemisorption [56]. To simplify the data analysis, only the enthalpy and entropy for that reaction are optimized. The optimization procedure for those terms uses a gradient search method by comparing the simulated results against the data collected at different temperatures for the AF160 material, and then iteratively changing the free energy term at each temperature to best describe the data. AF160 data are used for the optimization because the AO/IDO ligand ratio on the material is known [56].

**Figure 1:**
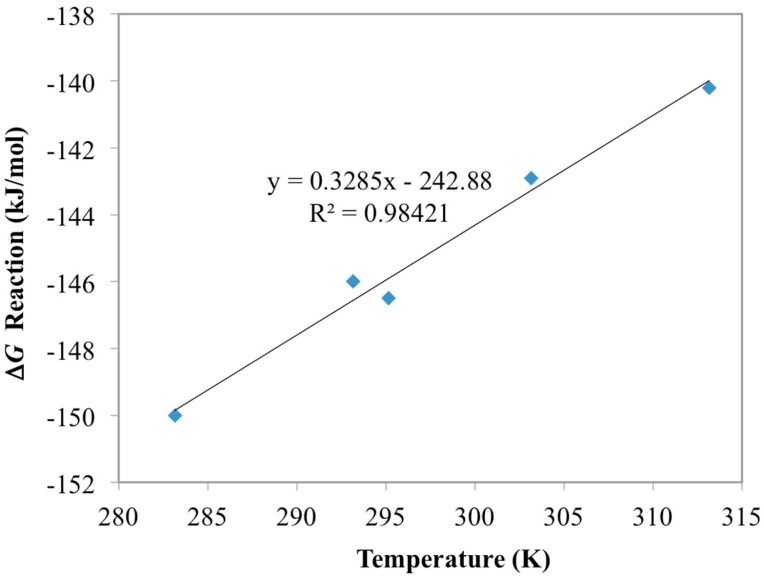
Optimum values for the free energy of the UO_2_(AO)_2_ forming reaction in Table 3. These values were obtained iteratively through comparison with the AF160 experimental data using a gradient search method.

**Figure 2:**
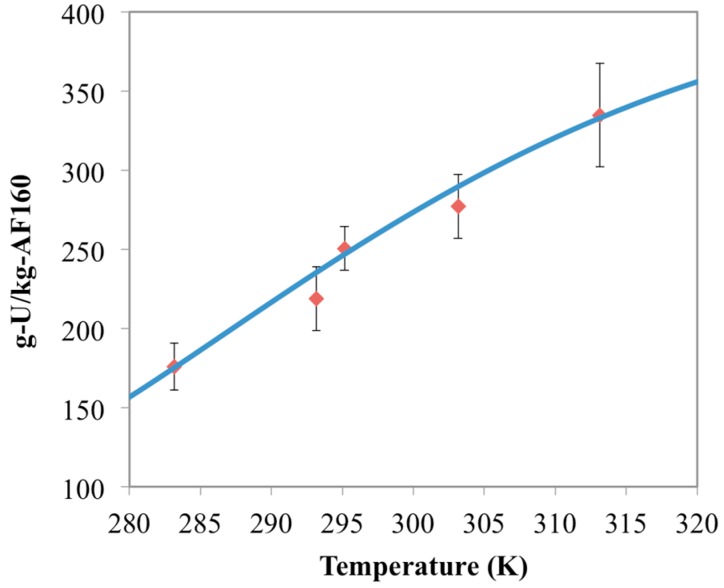
Comparison between optimized chemisorption model results (blue line) and the AF160 experimental data (red diamonds) collected in this study. Error bars represent two standard deviations of the data.

Optimum free energy terms at each temperature from this analysis are shown in Figure 1. These results show a clear linear trend between free energy (Δ*G*) and temperature, which adheres to Equation (3). The slope and intercept from the linear regression of these optimal values yield estimates for the entropy and enthalpy of the reaction. These energy values are reported in Table 3 and can be used to simulate the temperature effect of the other materials for uranium uptake. The optimized model results for the AF160 simulations are shown in Figure 2 and compared against the experimental data.

### 3.2. Prediction of Uranium Uptake for AF1FR3

After having obtained estimates for the reaction energies associated with the primary AO ligand reaction, this information can be used to try to predict the temperature effect for the uranium chemisorption by other materials used in the experiments. The AF1FR3 material has the same formulation as the AF160 and should have the same AO/IDO ligand ratio and same surface concentration of ITA comonomers (Table 2). Therefore, the chemisorption model developed in this work should be able to predict the amount of uranium uptake by AF1FR3 as a function of temperature or any other system parameter, such as changes in uranium concentration or variations in pH.

**Figure 3:**
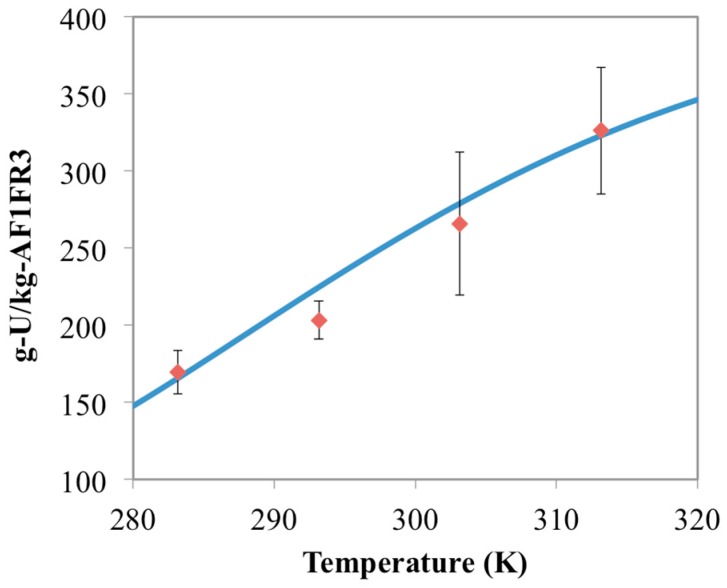
Comparison between chemisorption model results (blue line) and the AF1FR3 experimental data (red diamonds) for 15 mg of material. Error bars represent two standard deviations of the data.

**Figure 4:**
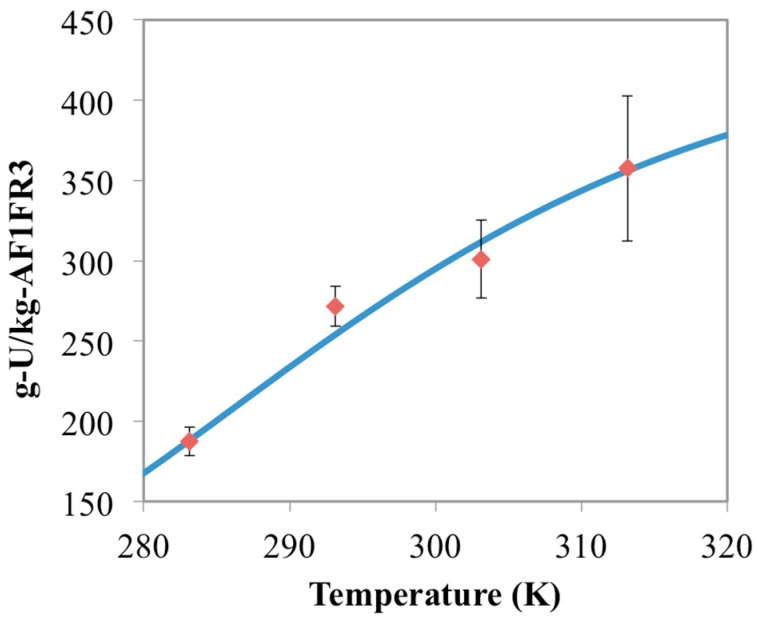
Comparison between chemisorption model results (blue line) and the AF1FR3 experimental data (red diamonds) for 10 mg of adsorbent material. Error bars represent two standard deviations of the data.

**Figure 5:**
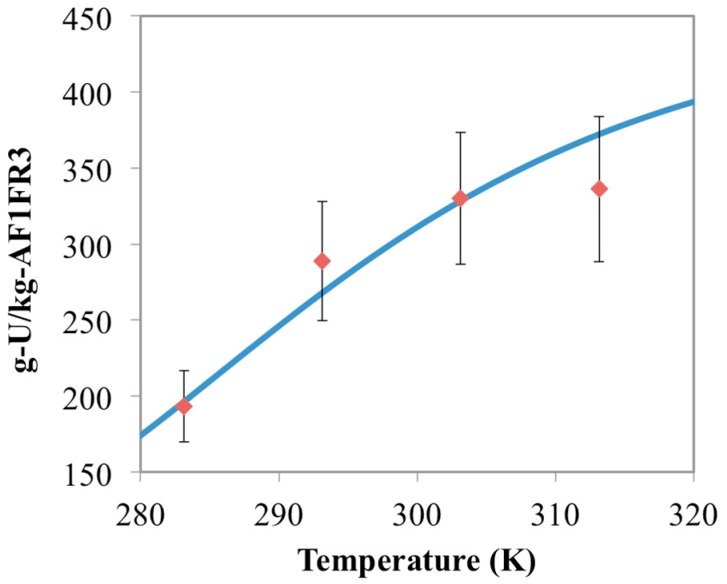
Comparison between chemisorption model results (blue line) and the AF1FR3 experimental data (red diamonds) for 5 mg of material. Error bars represent two standard deviations of the data.

Figure 3 shows the chemisorption data for the experimental runs with 15 mg of AF1FR3 at different temperatures compared to the simulated model results. As would be expected, both the simulated and experimental results are effectively the same as for the AF160 data. This is because AF160 and AF1FR3 have essentially the same material formulation, just prepared during different batches. To further evaluate the model, experiments with different masses of AF1FR3 were performed. The results from those experiments, as well as the model results, are shown in Figure 4 and Figure 5.

Results in Figure 4 show experimental data for 10 mg of AF1FR3, while Figure 5 shows the results with 5 mg of AF1FR3. Both the model and the data show slight increases in adsorption capacity as the mass of adsorbent inside the batch system decreases. This is because, as the mass of adsorbent decreases, there is more uranium left in solution. Thus, the equilibrium concentration of aqueous uranium is higher in the batches containing less adsorbent, which results in higher adsorption capacities. Less adsorbent also means that less uranium is captured overall. For instance, at 283 K, 15 mg of AF1FR3 had a capacity of 169 g-U/kg-adsorbent (Figure 3) while 5 mg of AF1FR3 had a capacity of 193 g-U/kg-adsorbent (Figure 5), but the total amount of captured uranium was 2.54 mg-U for 15 mg of material and 0.97 mg-U for 5 mg of material.

In addition to the model agreeing with the general trends seen in the data for AF1FR3, it also closely follows the magnitude of adsorption for all experimental cases (Figure 3 through Figure 5). This result helps to validate not just the modeling approach outlined in this work, but also the model parameters used to describe the system. From this validation, the model employed in this research may be used to predict how future amidoxime-based polymer adsorbents perform for uranium capture as a function of temperature, AO/IDO ligand ratio, type of comonomer, and other environmental factors such as pH and salinity.

### 3.3. Optimization of AO/IDO Ratio for the 38H Material

For the AF1 materials examined so far in this work, the AO/IDO ligand ratio was a known value based on prior experimental and modeling work [56]. However, while the total molar concentration of nitrogen is known for 38H and AI8 (Table 2), the amount of nitrogen that is either AO or IDO is unknown. Thus, the ratio of those two active ligands for 38H and AI8 is a missing piece of information needed to model and predict the uptake of uranium for these materials. Therefore, instead of trying to predict uranium uptake by those materials, the ratio of AO to IDO ligands can be estimated by simulating across all possible AO/IDO ratios that adhere to the nitrogen balance in Equation (1) and finding the simulation profile that best matches the experimental data.

**Figure 6:**
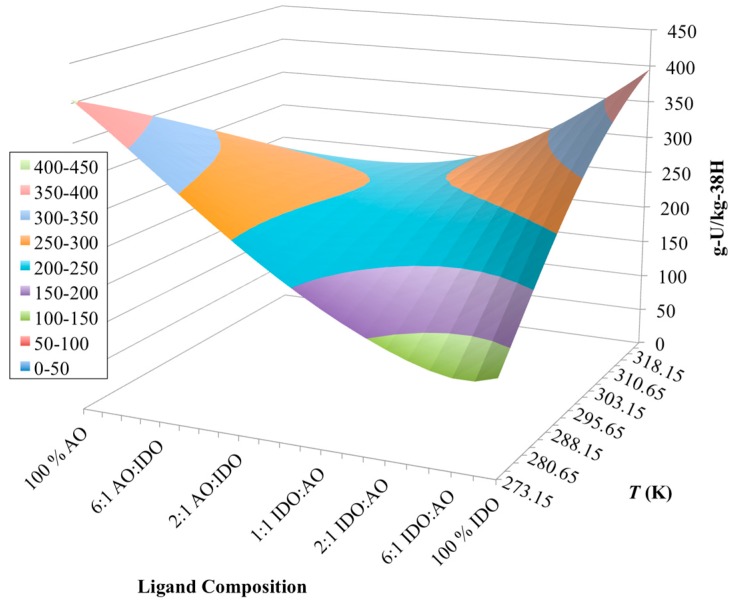
Simulation results of the chemisorption model for various ligand compositions on the 38H material. Chemisorption of uranium appears to change from exothermic to endothermic as the ligand composition changes from 100% AO to 100% IDO.

Figure 6 shows the results of a series of simulations for the 38H material at different temperatures and different ligand compositions. The ligand composition was varied from 100% IDO (*L*_max,IDO_ = 3.29 mol-IDO/kg-38H) to 100% AO (*L*_max,AO_ = 4.93 mol-AO/kg-38H), while ensuring that the nitrogen balance (Equation 1) was never violated. Results from these simulations reveal a “saddle” shaped contour for the uranium capacity, with maximum values occurring at the highest temperature for 100% IDO and lowest temperature for 100% AO. This result would imply that the behavior of the 38H material swaps from exothermic for 100% AO ligands to endothermic for 100% IDO ligands.

By examining the enthalpies for uranyl chemisorption reactions in Table 3, it can be seen that all enthalpies considered by the model are negative, which would imply that uranyl chemisorption was always exothermic, regardless of the ligand composition. However, it was noted by Leggett et al. [65] that the overall reaction for the IDO ligands is indeed endothermic. This was determined by also examining the enthalpies associated with the formation of the uranyl-tricarbonate species [UO_2_(CO_3_)_3_^4−^], which is the most prevalent aqueous uranium species in solution [2,68]. Collectively, the reaction enthalpy between UO_2_(CO_3_)_3_^4−^ and IDO was found to be endothermic [65], which is also shown by the simulation results from this chemisorption model (Figure 6).

For the AO reactions with uranium, the optimized enthalpy for the reaction forming UO_2_(AO)_2_ was found to be notably more negative than those of the IDO and uranyl reactions (Table 3). This observation means that that AO reaction is significantly less favorable at higher temperatures than the reactions with the IDO ligand, which may explain why the overall impact of uranyl chemisorption on AO shows itself to be exothermic (Figure 6). It is worth noting, however, that the enthalpy for the formation of UO_2_(AO)_2_ found in this study was estimated iteratively, ignoring the enthalpies of other AO reactions, and not actually measured. Thus, this estimate is only good as an effective enthalpy of reaction between uranyl and AO ligands.

**Figure 7:**
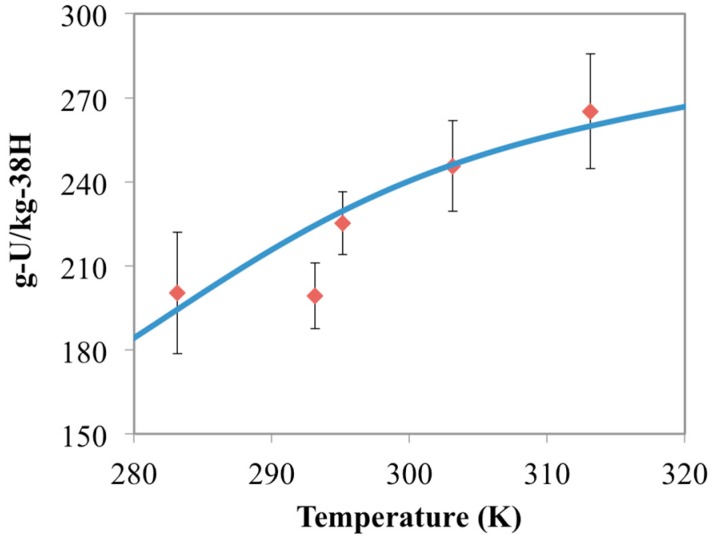
Comparison between chemisorption model results (blue line) and the 38H experimental data (red diamonds) collected in this study. Error bars represent two standard deviations of the data.

After having simulated uranium chemisorption by 38H across a wide range of ligand compositions, this information can be used to attempt to determine the AO/IDO ratio of 38H based on comparisons between model results and experimental data. Through that comparative analysis, it was determined that the molar concentration of AO and IDO were approximately 1.72 mol-AO/kg-38H and 2.14 mol-IDO/kg-38H, respectively. The simulation results at this surface composition are shown in Figure 7. Here, it is clear to see that the model compares well with the experimental data at all but one point; however, this point also does not fall in line with the general trend shown by the data.

### 3.4. Optimization of AO/IDO Ratio for AI8 Material

**Figure 8:**
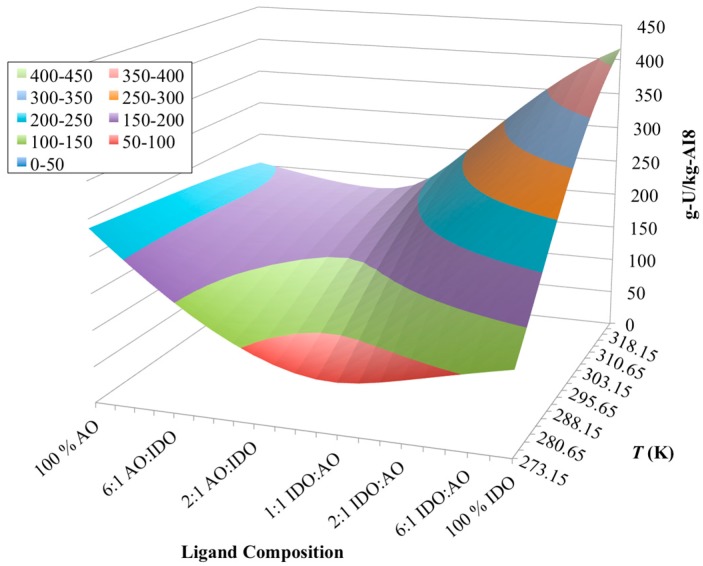
Simulation results of the chemisorption model for various ligand compositions on the AI8 material. Chemisorption of uranium appears to change from slightly exothermic to endothermic as the ligand composition changes from 100% AO to 100% IDO.

As shown before with the 38H material, the AO/IDO ratio of ligands for AI8 is also unknown. Therefore, uranium uptake simulations with AI8 were carried out over the range of AO and IDO surface concentrations that were applicable according to the estimated nitrogen concentration from Table 2 and the nitrogen balance in Equation 1. Abiding by the nitrogen balance, this would mean that the range of ligand concentrations varied from 4.36 mol-AO/kg-AI8 for 100% AO ligands and 2.90 mol-IDO/kg-AI8 for 100% IDO ligands. Results of the simulations across the spectra of AO/IDO ratios are shown in Figure 8.

Figure 8 shows that the reaction between uranium and the IDO ligand is endothermic (i.e., capacity increases with increasing temperature), as before with the 38H and AF1 materials. Additionally, the reaction with the AO ligand does appear to be exothermic, but the impact is significantly reduced from the simulations with 38H (Figure 6). No longer does the contour plot appear “saddle” shaped as before, and instead flattens out as the ligand composition moves towards 100 % AO. Since the simulation cases between 38H and AI8 have nearly the same mass of material, uranium concentration, and total nitrogen surface concentrations, it is very unlikely that any minor differences between these conditions would cause the effect shown in Figure 8. The only major difference between 38H and AI8 is the type and amount of the comonomers (Table 2). Thus, the comonomers must be controlling the major changes seen in the simulation plots for 38H (Figure 6) to AI8 (Figure 8).

In the chemisorption model developed in this work, the comonomers do not become directly involved with the binding of uranyl ions from solution. They will only undergo protonation and/or deprotonation reactions according to their p*K_a_*s and/or free energies (Table 3). However, since the comonomers are treated as part of the surface of the adsorbent, these protonation reactions will impact the overall charge accumulation for the material. This accumulation of charge will in turn have an indirect impact on the binding affinity for the chemisorption reactions through the Boltzmann factor (Equations (7) and (8)).

The 38H material contains the MAA comonomer, which is a monoprotic acid, while the AI8 uses VPA, which is diprotic. As such, the AI8 material would have a higher tendency to accumulate a larger amount of negative surface charge than 38H. The more negative charge that a surface gains, the more favorable chemisorption of positively charged ions becomes. In other words, the chemisorption reactions forming species UO_2_(AO)^+^ and UO_2_(H_2_IDO)^+^ will begin to become relevant to the overall uranium uptake due to the local charge equilibrium condition of the Gouy-Chapman theory [60,67]. This consideration is especially important in terms of the chemisorption on the AO ligands, for which there is no known information regarding the enthalpies of chemisorption reactions (Table 3).

Furthermore, the chemisorption model discussed in this work uses enthalpies and entropies to calculate how the binding affinities change with temperature (Equations (2) and (3)). If those energy values are unknown, then the model uses the Gibb’s free energy term (Δ*G*) instead. While this approach may work well for small temperature ranges, as that temperature range expands, it will introduce more error. Essentially, using the free energy term alone would be mathematically equivalent to assuming the entropy change of the reaction is zero and the enthalpy were equal to the Gibb’s free energy. As such, this would mean that the effective enthalpy of the AO reaction forming UO_2_(AO)^+^ would be −77.6 kJ/mol, which is significantly larger than the enthalpy for UO_2_(AO)_2_, making the UO_2_(AO)^+^ reaction much less exothermic.

Based on these considerations, if the AI8 material is accumulating more negative surface charge than 38H from the use of the VPA comonomer, then, as the ligand composition moves towards 100% AO ligands, the reaction forming UO_2_(AO)^+^ will become of greater importance to uranium uptake. In addition, if that reaction is much less exothermic than the reaction for UO_2_(AO)_2_, then the temperature effect on the chemisorption of uranium by AO ligands would be dramatically reduced (i.e., changes in uranium capacity with temperature would be much less pronounced), as shown in Figure 8. This analysis helps to explain the disappearance of the saddle-like shape of the contour plot from 38H (Figure 6) and AI8 (Figure 8) simulations.

**Figure 9:**
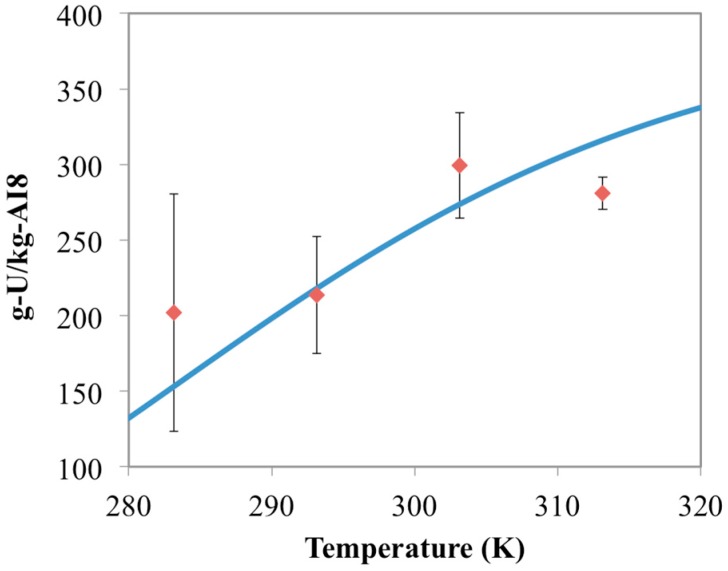
Comparison between chemisorption model results (blue line) and the AI8 experimental data (red diamonds) collected in this study. Error bars represent two standard deviations of the data.

Using the contours of uranium uptake from AI8 (Figure 8), the ligand ratio of AO to IDO can be determined by comparing each profile to the experimental data collected for AI8 in this work. Similarly to the work with the 38H data, each of the simulation profiles for AI8 was compared to the experimental data, and this analysis found that the ligand concentrations that best corresponded to the experimental data were 0.87 mol-AO/kg-AI8 and 2.32 mol-IDO/kg-AI8. The simulation results at these ligand concentrations compared to the data are shown in Figure 9. These results indicate that the model follows the trends observed in the data, but there is significantly more error in these results than in the other model comparisons. Some of this error may be attributable to noise in the experimental data that resulted in large standard deviations for the recorded adsorption capacities, but there is also uncertainty in the model due to gaps in information, such as missing enthalpies and entropies for several chemisorption reactions (Table 3).

**Figure 10:**
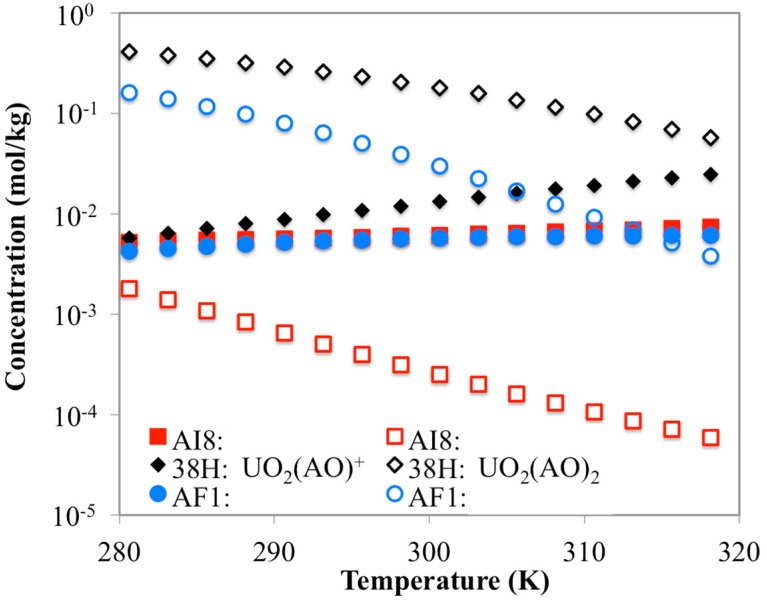
Simulated concentrations for UO_2_(AO)^+^ (filled shapes) and UO_2_(AO)_2_ (hollow shapes) for all three materials. While simulations with AF160 and 38H materials have shown UO_2_(AO)_2_ to be dominant, these results indicate that the AI8 material has UO_2_(AO)^+^ as the dominant uranyl-AO species.

If the speciation of uranium chemisorption on the AI8 material is observed more closely at the same ligand ratio (0.87 mol-AO/kg-AI8 and 2.32 mol-IDO/kg-AI8), it can be seen that the UO_2_(AO)^+^ species does become the dominant adsorbed uranium surface species for the AO ligands (Figure 10). This is a result that is not observed with the AF1 or 38H materials, and helps to further support the conclusion that the disappearance of the “saddle” shape in the contour plots from 38H (Figure 6) to AI8 (Figure 8) may be caused by excessive accumulation of negative surface charge. Since 38H and AI8 materials have very similar ligand/nitrogen concentrations (Table 2), the major contributor to the surface charge accumulation is probably caused by the differences in the comonomers. Thus, the comonomers likely play an indirect role in uranium recovery.

Figure 10 also highlights a shortcoming of the current state of the model’s parameter knowledge that is caused by gaps in information regarding the actual enthalpies of the AO reactions. When these enthalpies are unknown, it can introduce error in the simulation results, especially as temperature is varied over a larger range. By missing information on the actual enthalpies of the AO reactions, which are needed to accurately portray the temperature effect of the chemisorption reactions, the simulation results may be incomplete or misleading. Thus, it is plausible that the contour plot for AI8 (Figure 8) is an artifact of incomplete parameter information.

## 4. Conclusions

All experimental data presented in this work showed that the ORNL amidoxime-based adsorbents have a tendency to produce higher adsorption capacities as temperature increases, thus indicating that uranium chemisorption is endothermic. While this result seems inconsistent with the negative enthalpies of the chemisorption reactions examined, it is a result that has been reproduced in many other experiments both in laboratory and marine/oceanic settings [72,73,74]. Additionally, it has been determined that the overall reaction between the uranium species in solution [UO_2_(CO_3_)_3_^4−^] and the protonated amidoxime ligands is actually endothermic [65]. This result was also accurately predicted by the chemisorption model described in this work.

A model analysis of the AF160 data produced estimates for the enthalpies and entropies of the chemisorption reaction that generates the UO_2_(AO)_2_ species with the AO ligand. This analysis was limited to adjusting the free energies of this reaction alone since it was previously identified as the only major contributing AO chemisorption reaction for AF160 near neutral pH [56]. Later simulations with other materials, notably AI8, however, indicate that the positively charged UO_2_(AO)^+^ species starts to play a more important role in chemisorption when negative surface charge begins to accumulate. Because of this result, it becomes paramount to determine the enthalpy of this reaction in order to accurately portray its behavior across a wider temperature range with other materials.

It was not possible to estimate the enthalpy for the UO_2_(AO)^+^ reaction from the adsorption data gathered in this work for two primary reasons: (i) the UO_2_(AO)^+^ species showed no appreciable chemisorption for the AF160 material, and therefore lacked any sensitivity when comparing simulation results to experimental data and (ii) the AI8 material, which was sensitive to UO_2_(AO)^+^, had an unknown AO/IDO ligand ratio, and thus there were too many parameters that could have been adjusted or changed to accurately describe the data. In other words, if the AI8 data were used to estimate both the enthalpy for the UO_2_(AO)^+^ reaction and the AO/IDO ligand ratio, it would be highly likely to produce a false result since there would be multiple enthalpies and AO/IDO ratios that could produce the same or similar chemisorption profiles. This observation highlights the need to determine those reaction enthalpies through experiments.

One of the more interesting aspects of the chemisorption model developed in this work is the ability to include comonomers as part of the simulation case. Until now, no other uranium recovery model has been able to include the impact of the comonomers to uranium uptake. Even though these comonomers do not become directly involved in binding of the metal ions, they do have impacts on metal-ion chemisorption. Those impacts are caused by the accumulation of surface charges while the comonomers undergo their own protonation/deprotonation reactions. The potential magnitude of the importance of the comonomer to the overall uranium uptake was demonstrated in the contour plots of the 38H (Figure 6) and AI8 (Figure 8) materials, which shows dramatic difference in chemisorption profiles, even though 38H and AI8 have very similar concentrations of active ligands (Table 2). Without inclusion of the indirect impacts of the comonomers on chemisorption in the model, the contour plots for 38H and AI8 would have displayed very similar uptake profiles.

Although there are some shortcomings in the model, those limitations seem to be primarily in current gaps in the fundamental thermodynamic knowledge of specific reactions. Understanding this restriction may help to aid future studies in uranium-amidoxime binding and steer research towards exploration of the thermodynamics of potential reaction pathways. Since the modeling framework developed here is able to incorporate the impact of temperature on uranium chemisorption, all that is needed are those missing system parameters to provide additional insight on the effect of temperature on uranium chemisorption. In addition, in spite of those gaps in parameter information, the results of the simulations for all materials do show good agreement with all the experimental data and across all observed temperatures. Therefore, the model in its current state may be adequate for the temperature range of interest, which in seawater testing has varied from 8 °C to 31 °C [75].

Since the U.S. Department of Energy began funding research into the development of advanced amidoxime adsorbent materials, there has been a more than three-fold increase in the capacities of these materials for extracting uranium from seawater [9]. While this tremendous progress has helped to advance our understanding of the mechanisms of uranium extraction, it has still yet to produce a material that could economically compete with conventional mining for uranium recovery. Current best estimates of the cost of uranium production from seawater with these amidoxime materials is near $600/kg-U [46], which is much higher than the current value of uranium (~$60 –100/kg-U) [76]. Thus, further advancement in the capacity, selectivity, and durability of the materials is needed to make these adsorbents suitable for uranium recovery from natural seawater.

## Figures and Tables

**Table 1:** Elemental analysis results for the 38H adsorbent material before and after grafting of copolymers and after amidoximation.

Sample ID	Primary Elements (wt %)			
	C	H	N	O
PE hollow gear fiber	84.9	14.5	<0.5	<0.5
Grafted 38H	62.3	7.8	12.6	15.8
Amidoximated 38H	49.9	7.2	13.8	24.0

**Table 2:** Summary of nitrogen and comonomer surface concentrations after preparation of sorbent materials.

Adsorbent	Comonomer	N Concentration (mol/kg)	Comonomer Concentration (mol/kg)
38H	MAA	9.9	5.5
AI8	VPA	8.7 *	2.5 *
AF1	ITA	12.8	2.0

* AI8 material surface composition is estimated only from DOG% and AN/VPA ratio of grafting solution. No elemental analysis was available.

**Table 3:** Summary of uranyl/ligand reactions considered by the chemisorption model. All Δ*G* values are given at 25 °C.

Chemisorption Reactions	*ΔG* (kJ/mol)	*ΔH* (kJ/mol)	*ΔS* (J/K/mol)
**Acetamidoxime (AO):**			
UO_2_^2+^ + AO^−^ ⇆ UO_2_(AO)^+^	−77.6 ^a^		
UO_2_^2+^ + 2AO^−^ ⇆ UO_2_(AO)_2_	−135.3 ^a^	−242.9 ^b^	−329 ^b^
UO_2_^2+^ + 3AO^−^ ⇆ UO_2_(AO)_3_^−^	−159.3 ^c^		
UO_2_^2+^ + AO^−^ + CO_3_^2−^ ⇆ UO_2_(AO)(CO_3_)^−^	−90.2 ^c^		
UO_2_^2+^ + 2AO^−^ + CO_3_^2−^ ⇆ UO_2_(AO)_2_(CO_3_)^2−^	−145.6 ^c^		
**Glutarimide-Dioxime (IDO):**			
UO_2_^2+^ + HIDO^2−^ ⇆ UO_2_(HIDO)	−109.6 ^d^	−67.3 ^d^	142 ^d^
UO_2_^2+^ + H^+^ + HIDO^2−^ ⇆ UO_2_(H_2_IDO)^+^	−134.1 ^d^	−75.4 ^d^	197 ^d^
UO_2_^2+^ + 2HIDO^2−^ ⇆ UO_2_(HIDO)_2_^2−^	−165.5 ^d^	−109.0 ^d^	188 ^d^
UO_2_^2+^ + H^+^ + 2HIDO^2−^ ⇆ UO_2_(H_2_IDO)(HIDO)^−^	−222.0 ^d^	−130.0 ^d^	309 ^d^
UO_2_^2+^ + 2H^+^ + 2HIDO^2−^ ⇆ UO_2_(H_2_IDO)_2_	−252.3 ^d^	−161.0 ^d^	307 ^d^
UO_2_^2+^ + HIDO^2−^ + CO_3_^2−^ ⇆ UO_2_(HIDO)(CO_3_)^2−^	−143.8 ^c^		
UO_2_^2+^ + H^+^ + HIDO^2−^ + CO_3_^2−^ ⇆ UO_2_(H_2_IDO)(CO_3_)^−^	−166.7		

^a^ Taken from refs. [63,64] and corrected to zero ionic strength with the Davies model [66]; ^b^ Determined via optimization with AF160 data in this study; ^c^ Taken from refs. [47,56]; ^d^ Taken from ref. [65] and corrected to zero ionic strength with the Davies model [66].

**Table 4:** Summary of p*K_a_*s and free energies of reaction for the active surface ligands and comonomers [52,53,63,64,68,69,70,71]. All values are reported at 25 °C unless otherwise noted.

Protonation/Deprotonation Reaction	p*K*_a_	Δ*G* (kJ/mol)
H_2_AO^+^ ⇆ HAO + H^+^	5.8	33.0
HAO ⇆ AO^−^ + H^+^	13.2	75.4
H_4_IDO^+^ ⇆ H_3_IDO + H^+^	2.1	12.1
H_3_IDO ⇆ H_2_IDO^−^ + H^+^	11.0	63.4
H_2_IDO^−^ ⇆ HIDO^2−^ + H^+^	12.9	73.3
HMAA ⇆ MAA^−^ + H^+^	4.7	26.2 *
H_2_VPA ⇆ HVPA^−^ + H^+^	2.7	15.4 *
HVPA^−^ ⇆ VPA^2−^ + H^+^	7.3	41.2 *
H_2_ITA ⇆ HITA^−^ + H^+^	3.9	22.0
HITA^−^ ⇆ ITA^2−^ + H^+^	5.5	31.1

* Reference temperature of 20 °C.

## Supplementary Materials

The following are available online at http://www.mdpi.com/1996-1944/10/11/1268/s1.
